# The complete chloroplast genome sequences of *Zanthoxylum nitidum* var. *nitidum* and *Z. nitidum* var. *tomentosum*

**DOI:** 10.1080/23802359.2019.1688113

**Published:** 2019-11-13

**Authors:** Yunrui Qin, Guilan Dang, Guiyuan Wei, Chuanggui Xu, Yunfeng Huang

**Affiliations:** a Guangxi Institute of Chinese Medicine and Pharmaceutical Sciences, Nanning, Guangxi, China;; b Guangxi Key Laboratory of Traditional Chinese Medicine Quality Standards, Guangxi Institute of Chinese Medicine & Pharmaceutical Sciences, Nanning, Guangxi, China

**Keywords:** *Zanthoxylum nitidum*, chloroplast genome, phylogenetic analysis

## Abstract

*Zanthoxylum nitidum* (Rutaceae Juss.) is a traditional Chinese medicine with four morphological types. We assembled their complete chloroplast genome sequences. The assembled genomes are 156,999–157,349 bp in length, including a large single copy (LSC) region of 84,064–84,455 bp, a small single copy (SSC) region of 17,582–17,651 bp and a pair of inverted repeats (IRs) of 27,631–27,659 bp. All genomes contained 133 genes, including 79 protein-coding genes, 30 tRNA genes, and four rRNA genes. The GC content is 38.5%. The further phylogenetic analysis showed that *Z. nitidum* clade was a monophyletic group separated from other *Zanthoxylum* species.

*Zanthoxylum nitidum* (Rutaceae Juss.) as a famous traditional Chinese medicine contains two varieties, corresponding to *Z. nitidum* var. *nitidum* and *Z. nitidum* var. *tomentosum*. The former is further divided into three types according to the diversified morphological traits (Huang 1997; Qin et al. 2019). The plastid genome will contribute to develop DNA barcodes for the rapid recognition and classification of *Z. nitidum.* Here, we report the plastid genomes of *Z. nitidum* (including two varieties and four morphological types) clarify their phylogenetic positions.

Fresh leaves were collected from *Z. nitidum* var. nitidum (Type 1: N23°12′10″, E110°12′12″, 478 m; Type 2: N25°01′03′, E107°09′42′, 535 m; Type 3: N22°57′1″, E106°43′52″, 270 m) and *Z. nitidum* var. *tomentosum* (N110°11′29″, E24°06′53″, 914 m) in Guangxi, China. All the voucher specimens (*Yunfeng Huang 20170519001*, *Yunfeng Huang 20180914005*, *Yunfeng Huang 20180814006* and *Yunfeng Huang 20170504015*) were deposited in GXMI. The total DNA was extracted using the modified CTAB method (Doyle 1987). DNA data were sequenced using the Illumina HiSeq 2500 platform. Subsequently, chloroplast genome assembly using GetOrganelle (Jin et al. 2018) and the annotation was performed with PGA (Qu et al. 2019). The other detailed process referred to Xiang et al. (2019). Finally, the complete chloroplast genome was deposited in GenBank (accessible numbers: MN241095, MN241096, MN241097, and MN241098).

Four complete chloroplast genomes of *Z. nitidum* var. *nitidum* (Type 1–Type 3) and *Z. nitidum* var. *tomentosum* was 157,349 bp, 156,999 bp, 157,217 bp, and 157,031 bp, respectively. They exhibit the typical quadripartite structure, including a pair of inverted repeats (IRs) (27,651 bp, 27,642 bp, 27,655 bp, and 27,659 bp, respectively) separated by the large single copy (LSC) (84,413 bp, 84,064 bp, 84,275 bp, and 84,455 bp, respectively) and small single copy (SSC) (17,634 bp, 17,651 bp, 17,632 bp, and 17,582 bp, respectively) regions. Each of them contains 113 unique genes, of which 20 were duplicated in the IRs, including 79 protein coding genes, 30 unique tRNA genes, and four unique rRNA genes. Among the annotated genes, 16 of them contained one intron, and two genes (ycf3 and clpP) included a couple. These four morphological types of *Z. nitidum* have the same base compositions of the whole chloroplast genome, with an overall GC content of 38.5%.

To further confirm their phylogenetic positions, we used RAxML to construct a maximum likelihood tree using 1000 bootstraps under the GTRGAMMA substitution mode (Stamatakis 2014). Sixteen complete chloroplast genomes in Rutaceae and two outgroups were selected. Sequences were aligned using MAFFT v.7 (Katoh and Standley 2013). Our results suggested *Z. nitidum* var. *nitidum* and *Z. nitidum* var. *tomentosum* comprised a sister lineage and *Z. nitidum* clade was separated from other *Zanthoxylum* species (Figure 1).

**Figure 1.**
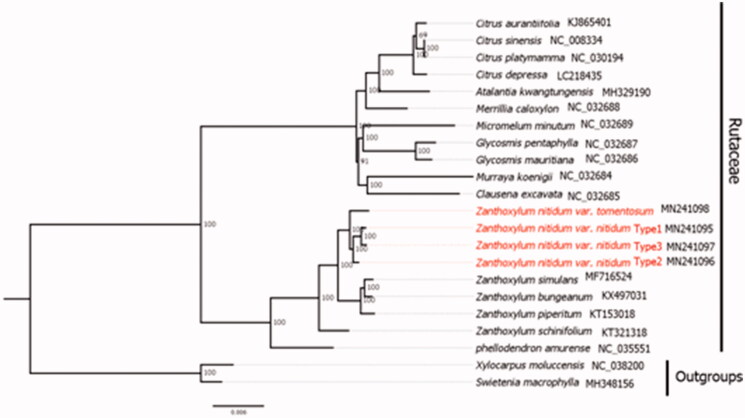
The best ML phylogeny recovered from 22 complete chloroplast genomes sequences by RAxML. NCBI accession number is shown after each species name.
